# An autopsied case report of spastic paraplegia with thin corpus callosum carrying a novel mutation in the *SPG11* gene: widespread degeneration with eosinophilic inclusions

**DOI:** 10.1186/s12883-021-02514-z

**Published:** 2022-01-03

**Authors:** Mika Hayakawa, Tomoyasu Matsubara, Yoko Mochizuki, Chisen Takeuchi, Motoyuki Minamitani, Masayuki Imai, Kenjiro Kosaki, Tomio Arai, Shigeo Murayama

**Affiliations:** 1 Department of Pediatrics, Tokyo Metropolitan Kita Medical and Rehabilitation Center for the Disabled, 1-2-3 Jujodai Kita-ku, Tokyo, 114-0033 Japan; 2 Department of Neurology and Neuropathology (the Brain Bank for Aging Research), Tokyo Metropolitan Geriatric Hospital and Institute of Gerontology, Tokyo, Japan, 35-2 Sakae-cho, Itabashi-ku, Tokyo, 173-0015 Japan; 3 Department of Neurology, Tokyo Metropolitan Kita Medical and Rehabilitation Center for the Disabled, 1-2-3 Jujodai Kita-ku, Tokyo, 114-0033 Japan; 4 Center for Medical Genetics, Keio University School of Medicine, 35 Shinano-machi, Shinjyuku-ku, Tokyo, 160-8582 Japan; 5 Department of Pathology, Tokyo Metropolitan Geriatric Hospital and Institute of Gerontology, 35-2 Sakae-cho, Itabashi-ku, Tokyo, 173-0015 Japan; 6 The Brain Bank for Neurodevelopmental, Neurological and Psychiatric Disorders, United Graduate School of Child Development, Osaka University, 2-2 Yamadaoka, Suita-shi, Osaka, 565-0871 Japan

**Keywords:** Alpha-synuclein, Case report, Hereditary spastic paraplegia with a thin corpus callosum, Lateral geniculate body, Lewy body-related α-synucleinopathy, p62-immunoreactive neuronal cytoplasmic inclusions, *SPG11* gene

## Abstract

**Background:**

The detailed neuropathological features of patients with autosomal recessive hereditary spastic paraplegia with a thin corpus callosum (TCC) and *SPG11* mutations are poorly understood, as only a few autopsies have been reported. Herein, we describe the clinicopathological findings of a patient with this disease who received long-term care at our medical facility.

**Case presentation:**

A Japanese man exhibited a mild developmental delay in early childhood and intellectual disability, followed by the appearance of a spastic gait by age 13. At the age of 25 years, he became bedridden and needed a ventilator. Genetic analysis revealed a homozygous splice site variant in the *SPG11* gene (c. 4162–2A > G) after the provision of genetic counselling and acquisition of informed consent from his parents. He died of pneumonia at the age of 44. His brain weighed 967 g and was characterized by a TCC, and his spinal cord was flattened. Microscopically, degeneration was observed in the posterior spinocerebellar tract, the gracile fasciculus, and the posterior column in addition to the corticospinal tract. Marked neuronal loss and gliosis were observed in the anterior horn, Clarke’s column, and hypoglossal and facial nuclei. Various types of neurons, in addition to motor neurons, showed coarse eosinophilic granules that were immunoreactive for p62. The loss of pigmented neurons with gliosis was apparent in both the substantia nigra and locus coeruleus. Lateral geniculate body degeneration was a characteristic feature of this patient. Furthermore, peripheral Lewy body-related α-synucleinopathy and scattered α-synuclein–immunoreactive neurites in the locus coeruleus and reticular formation of the brainstem were observed.

**Conclusions:**

In patients with hereditary spastic paraplegia with *SPG11* mutations, a variety of clinical phenotypes develop due to widespread lesions containing p62-immunoreactive neuronal cytoplasmic inclusions. We herein report the lateral geniculate body as another degenerative site related to *SPG11*-related pathologies that should be studied in future investigations.

**Supplementary Information:**

The online version contains supplementary material available at 10.1186/s12883-021-02514-z.

## Background

Autosomal recessive hereditary spastic paraplegia with a thin corpus callosum (ARHSP-TCC) is characterized by slowly progressive spastic paraparesis and intellectual disability and is reported to be a clinicopathologically unique complicated form of HSP in studies on Japanese patients [1, 2]. The genetic causes of ARHSP-TCC have been incrementally elucidated. In 1999, Martinez et al. performed genetic linkage analysis, and the results suggested a new locus for ARHSP linked to chromosome 15q13–15 in families with ARHSP-TCC from America and Europe; this locus was termed *SPG11* because it was the eleventh gene discovered to underlie HSP [3]. Later, Shibasaki et al. reported 10 Japanese ARHSP-TCC families, including two previously reported families [2, 4], who also showed linkage to the 15q13–15 locus [5]. After that, Stevanin et al. analysed HSP with *SPG11* mutations in index patients and identified mutations in the *KIAA1840* gene, which encodes spatacsin [6]. From these findings, *SPG11* mutations are now widely known as the most common cause of ARHSP-TCC [6–12]. However, the detailed neuropathological features of ARHSP-TCC with *SPG11* mutations are poorly understood due to the limited number of autopsy reports [13–15].

Here, we describe the clinical and pathological characteristics of an autopsied patient with spastic paraplegia with a TCC and a homozygous splice site variant in the *SPG11* gene (NM_025137.3: c.4162–2A > G).

## Case presentation

**Fig. 1:**
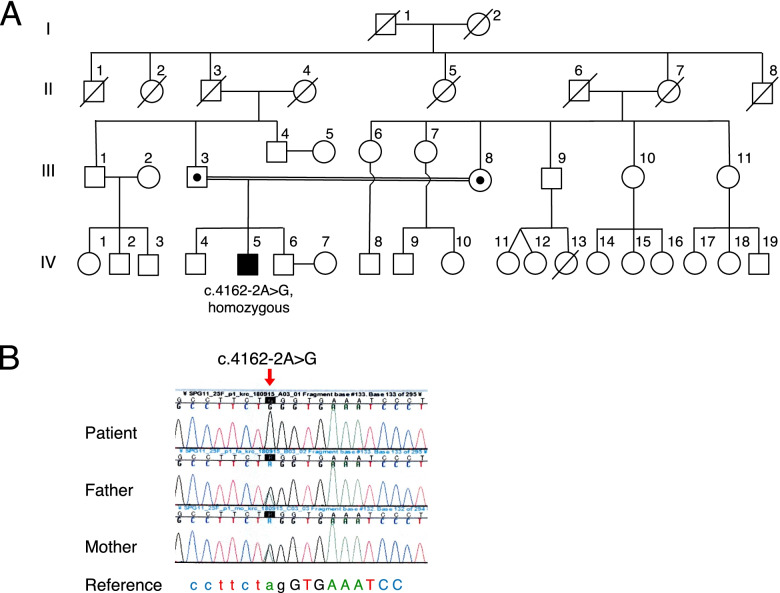
**A** Pedigree of the family. The proband (IV-5) had a homozygous variant of *SPG11*. The parents (III-3 and III-8) were cousins. **B** Electropherogram of *SPG11* variants. A homozygous splice site variant of c.4162–2A > G was identified in the patient. A heterozygous variant at the same location was identified in the parents. The location of the variant is shown by the red arrow

The patient was a Japanese man, and his parents were consanguineous (Fig. 1A). He was born full-term via vaginal delivery. He exhibited a mild developmental delay in early childhood and was diagnosed with an intellectual disability at 8 years old. Gait disturbance with an equinovarus foot deformity and leg spasticity was observed at the age of 13 years. At the age of 19, he was admitted to our hospital. At that time, his IQ was 35 based on the Tanaka-Binet Intelligence Scale. His eye movements were saccadic without nystagmus, and he had mild dysarthria. He showed muscle weakness in both lower limbs and a spastic gait. He did not have bradykinesia, muscle rigidity, cerebellar ataxia, or sensory disturbances. Magnetic resonance imaging of the brain showed global thinning of the corpus callosum, a wide cavum verge and cavum septi pellucidi, an abnormally high area of intensity in the periventricular white matter, and hypoplasia or atrophy of the frontal lobe [16]. Based on these findings, he was diagnosed with HSP with a TCC.

**Fig. 2:**
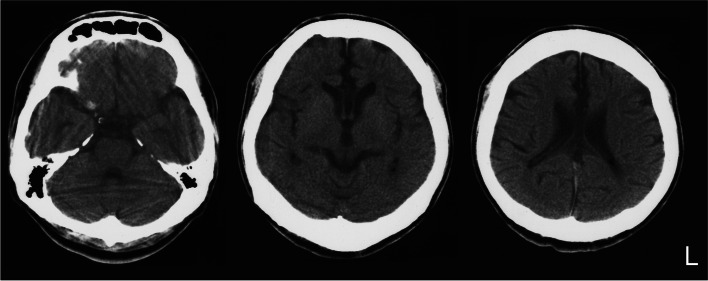
Computed tomography of the patient’s brain at the age of 24 years. The skull was thick, and the corpus callosum showed thinning. The frontal and temporal lobes were atrophic, while the cerebellum was relatively preserved

At the age of 20 years, he developed clumsiness and weakness in the upper limbs. The following year, he was not able to walk by himself. At the age of 23 years, nerve conduction studies showed mild delays in the conduction velocities in the motor nerves of the upper and lower limbs and in the sensory nerves of the upper limbs. Atrophy of his frontal and temporal lobes was revealed at the age of 24 years by brain computed tomography (Fig. 2). At the age of 25 years, he became bedridden, dysarthria progressed, and dysphagia and a depressive state appeared. Therefore, he required feeding by a nasal tube. One month later, he underwent sudden respiratory arrest and was thus intubated and required a ventilator. Respiratory arrest occurred while his arterial oxygen saturation of pulse oxymetry and heart rate were monitored; therefore, he did not have cardiac arrest, and his level of consciousness did not change before and after the event. One month later, his spontaneous breathing recovered, but due to unstable respiratory functioning, he underwent a tracheostomy. At the age of 39 years, he became quadriplegic, with absent deep tendon reflexes and distal dominant muscle amyotrophy, and he required permanent ventilation from this point forward. Ocular fixation was observed, but his pupil light reflex was delayed. He died of pneumonia at the age of 44 years, 31 years after the onset of spasticity. He did not have seizures at any time during the course of his disease.

### Genetic analysis results

A detailed description of genetic analysis methods is provided in Additional file 1. TruSight One sequencing panels revealed a mutation around exon 25 of *SPG11*. Direct Sanger sequencing of *SPG11* revealed a homozygous variant of *SPG11* (c. 4162 − 2A > G) (Fig. 1B). A heterozygous variant at the same location was identified in both parents. This variant was located at the splice acceptor site of intron 24 of *SPG11* and was predicted to be a disease-causing (very strong pathogenicity, PVS1) mutation of a known causative gene of ARHSP-TCC according to the American College of Medical Genetics and Genomics standards and guidelines [17]. Furthermore, we could not find this variant in ClinVar (https://www.ncbi.nlm.nih.gov/clinvar/), the Japanese genome database of Human Genetic Variation (http://www.hgvd.genome.med.kyoto-u.ac.jp), or the Japanese Reference Genome version 1 (https://jmorp.megabank.tohoku.ac.jp/202001/variants).

### Pathological findings

An autopsy was performed 13.5 h after death. A detailed description of neuropathological examination methods is provided in Additional file 1. The patient had a right lung abscess and left focal aspiration pneumonia, and his cause of death was respiratory failure.

**Fig. 3:**
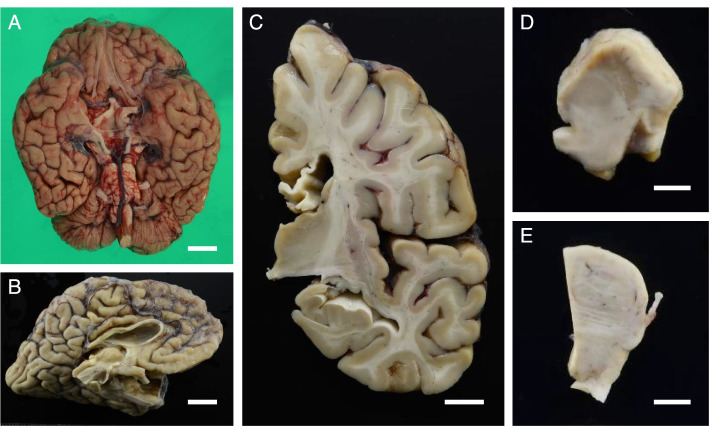
Macroscopic findings. **A** The brain was small overall. **B** In a sagittal section of the cerebrum, a thin corpus callosum and frontal lobe atrophy were observed. **C** In a coronal section, a thin corpus callosum, mild enlargement of the anterior horn of the lateral ventricle (resulting from decreased cerebral cortex and white matter volumes), and a mildly atrophic thalamus were observed. **D**, **E** The brainstem was small at all levels, and the substantia nigra and locus coeruleus were depigmented. Scale bars: 2 cm (**A**, **B**); 1 cm (**C**); 5 mm (**D**, **E**)

**Fig. 4:**
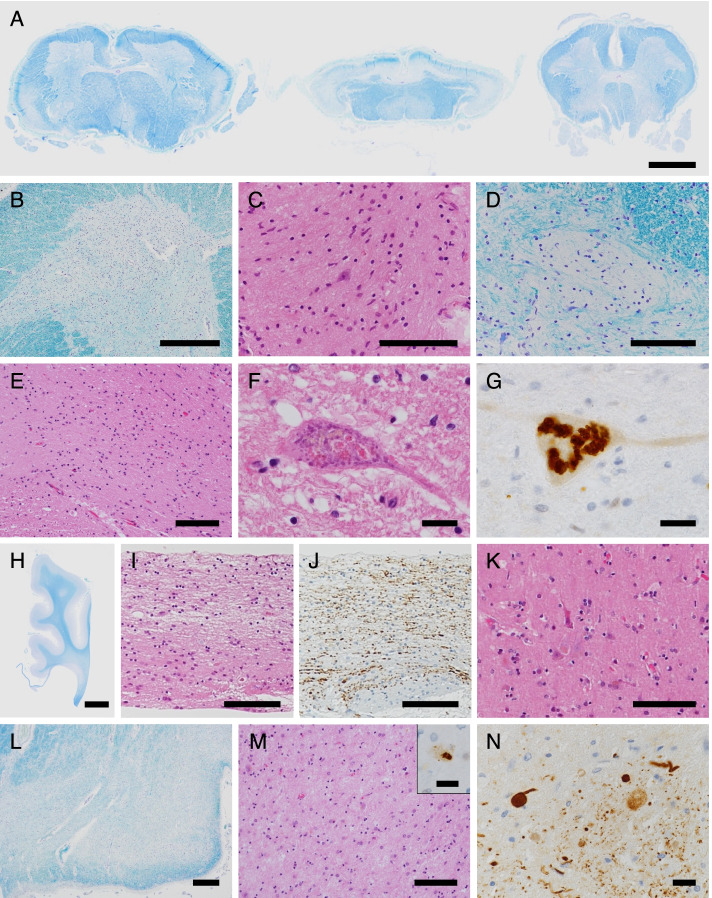
Microscopic findings. The spinal cord showed bilateral decreases in myelin staining in the corticospinal tract, the posterior spinocerebellar tract, the posterior column including the gracile fasciculus and the intermediate root zone, and the anterolateral funiculus (**A**). Marked neuronal loss and gliosis were observed in the spinal cord anterior horn (low-power field with Klüver-Barrera staining, **B**; high-power field with haematoxylin-eosin staining, **C**). Onuf’s nucleus (**D**) and Clarke’s column (**E**) also showed severe neuronal loss and gliosis. Coarse eosinophilic granules were observed in a cervical spinal cord anterior horn cell (**F**). These eosinophilic inclusions were immunoreactive for p62 (**G**). The corpus callosum was very thin (**H**) and exhibited gliosis, but slight axonal staining remained (**I**, **J**). The precentral gyrus showed moderate neuronal loss and gliosis, and Betz cells became so atrophic that they were difficult to identify (**K**). The lateral geniculate body showed marked neuronal loss and gliosis in all layers (low-power field with Klüver-Barrera staining, **L**; high-power field with haematoxylin-eosin staining, **M**; high-power field with p62 immunostaining, **M inset**), and Lewy body-related α-synucleinopathy was abundant in the dorsal motor nucleus of the vagus nerve (**N**). Scale bars: 2 mm (**A**); 500 μm (**B**, **L**); 100 μm (**C**–**E**, **I**–**K**, **M**); 20 μm (**F**, **G**, **M-Inset**, **N**); 5 mm (**H**)

In the brain, which weighed 967 g, the cerebrum, brainstem, and cerebellum were macroscopically small (Fig. 3A, B), and atrophy was particularly noticeable in the frontal lobe and the precentral gyrus (Fig. 3A, B). The corpus callosum was extremely thinned over the entire rostrocaudal length (Fig. 3B, C). The volumes of the cerebral cortex and white matter were decreased (Fig. 3C). The caudate nucleus and thalamus exhibited mild atrophy, and the thalamus was brownish in colour (Fig. 3C). In the brainstem, both the substantia nigra and locus coeruleus showed depigmentation (Fig. 3D, E). Atrophy of the optic nerve was not observed (Fig. 3A). The whole spinal cord was flattened, especially in the thoracic cord. Atrophy of the ventral predominant spinal roots was observed. Microscopically, in the spinal cord, the corticospinal tract, the posterior spinocerebellar tract, the posterior column (including gracile fasciculus and the intermediate root zone), and the anterolateral funiculus showed bilateral decreases in myelin and axonal staining (Fig. 4A). Marked neuronal loss and gliosis were observed in the anterior horn at all levels of the spinal cord (Fig. 4B, C). Moreover, Onuf’s nuclei and Clarke’s column also showed severe neuronal loss and gliosis (Fig. 4D, E), while the intermediolateral nuclei in the thoracic and sacral spinal cord regions were relatively spared. Severe neuronal loss and gliosis were also observed in the hypoglossal and facial nuclei, while the abducens and oculomotor nuclei were relatively preserved. Coarse eosinophilic granules were observed in the remaining neurons of all of these motor nuclei (Fig. 4F). These eosinophilic inclusions were immunoreactive for p62 and ubiquitin (Fig. 4G) but not for phosphorylated TAR DNA-binding protein 43 kDa or cystatin C. The p62-immunoreactive neuronal cytoplasmic inclusions (NCIs) were more numerous and widespread than the NCIs identified by haematoxylin-eosin staining. Table 1 shows the findings of neuronal loss, gliosis, and the frequency of p62-immunoreactive NCIs in various regions.

**Table 1:** Neuropathological findings

	Degeneration		p62-immunoreactive neuronal cytoplasmic inclusions
	Neuronal loss	Gliosis	Frequency
**Cerebral cortex**			
Frontal cortex	+	++	+
Motor cortex	++	++	+
Temporal cortex	-	+	+
Parietal cortex	-	+	+
Visual cortex	+	+	+
**Limbic areas**			
Amygdala	++	++	+
Hippocampus	+	+	-
Transentorhinal/entorhinal cortex	-	+	-
Anterior cingulate cortex	+	++	+
Insular cortex	+	++	-
**Basal ganglia**			
Caudate nucleus	++	++	+
Putamen	++	++	+
Globus pallidus	-	+	-
Subthalamic nucleus	+	+	+++
Nucleus basalis of Meynert	-	+	++
**Thalamus**			
Anterior nuclear group	++	+++	+
Dosal medial nuclear group	+++	+++	++
Ventral lateral nuclear group	+++	+++	++
Purvinar	+++	+++	+
Lateral geniculate body	+++	+++	+
**Midbrain**			
Oculomotor nucleus	-	+	+++
Red nucleus	+	++	-
Substantia nigra pars compacta	+++	++	++
Substantia nigra pars reticulata	++	++	+
**Pons**			
Trigeminal motor nucleus	+	++	+++
Facial nucleus	+++	+++	++
Pontine nucleus	-	+	+
Locus coeruleus	+++	+++	-
**Medulla oblongata**			
Hypoglossal nucleus	+++	++	++
Dorsal vagal nucleus	++	++	-
Inferior olivary nucleus	+	+	+
**Cerebellum**			
Purkinje cells in vermis	+++	+++	-
Purkinje cells in hemisphere	+	+	-
Cerebellar dentate nucleus	++	++	++
**Spinal cord**			
Anterior horn	+++	+++	+
Intermediate lateral nucleus	+	++	+
Clarke's column	++++	+++	-
Onuf's nucleus	++++	+++	-
**Peripheral tissue**			
Dorsal root ganglion	++	NA	+++

*NA* Not assessed

Neuronal loss was indicated as absent (-), mild (+), moderate (++), severe (+++), or totally lost (++++)

Gliosis was indicated as mild (+), moderate (++), or severe (+++)

The frequency of p62-immunoreactive neuronal cytoplasmic inclusions (NCI), evaluated at a 10× objective magnification, is indicated as follows: no NCI across the entire section (-), an average of 0–2 inclusions (+), an average of 3–9 inclusions (++), and an average of ≥10 inclusions (+++) per field

The medullary pyramid was atrophic and showed myelin and axonal loss, whereas the cerebral peduncle showed only a dimple in the middle. The loss of pigmented neurons with gliosis was apparent in both the substantia nigra and locus coeruleus. In the cerebellum, depletion of Purkinje cells with proliferation of Bergmann glia and isomorphic gliosis in the cerebellar white matter were predominantly observed in the vermis, and grumose degeneration—in addition to moderate neuronal loss and gliosis—was observed in the cerebellar dentate nucleus. The thalamus showed severe neuronal loss and gliosis. Moderate neuronal loss and gliosis were also observed in the striatum. The amygdala exhibited prominent gliosis; on the other hand, the hippocampus showed mild neuronal loss and gliosis. The rare appearance of p62-immunoreactive NCIs in the limbic areas was different from that observed in the other areas (Table 1). The corpus callosum was very thin (Fig. 4H) and accompanied by gliosis, but slight myelin and axonal staining remained (Fig. 4I, J). The neurons in the cerebral cortex appeared to be small, and the cortical widths seemed to be slightly narrow, especially in the frontal cortex; however, the layered structures were maintained. The precentral gyrus showed moderate neuronal loss and gliosis and a decreased number of radiating fibres. Betz cells became so atrophic that they were difficult to identify (Fig. 4K). To a lesser extent, the frontal lobe showed neuronal loss and gliosis. The volume of cerebral white matter was reduced, but some myelin staining remained. The lateral geniculate body (LGB) showed marked neuronal loss and gliosis in all layers (Fig. 4L, M), with secondary degeneration being observed in the optic radiation and the striate cortex. The optic nerve was well preserved. The sural nerve exhibited a severely decreased number of myelinated fibres, and many Nageotte nodules were observed in the spinal dorsal root ganglia.

As a comorbid pathology, Lewy body-related α-synucleinopathy (LBAS) was abundant in the peripheral nervous systems, including sympathetic ganglia, heart, skin, and gastrointestinal tract, dorsal motor nucleus of the vagus nerve, and solitary nuclei (Fig. 4N). Scattered α-synuclein–immunoreactive neurites were observed in the locus coeruleus and reticular formation of the brainstem. No LBAS was observed in the substantia nigra, basal ganglia, olfactory bulb, limbic systems, or cerebrum. Senile changes were minimal.

## Discussion and conclusions

Herein, we report an autopsied Japanese patient with HSP-TCC who had a novel mutation in the *SPG11* gene, which encodes spatacsin. The clinical phenotype of this patient was similar to that previously reported for HSP with mutations in *SPG11* [7].

In the present patient and other reported autopsied patients [13, 14], common pathological features include degeneration in the upper and lower motor neuron systems as well as in the grey and white matter of the cerebral neocortex, thalamus, substantia nigra, Clarke’s nuclei, spinocerebellar tract, sural nerve, and dorsal root ganglia. Furthermore, we described an LGB lesion with p62-immunoreactive NCIs for the first time in a patient with an *SPG11* mutation. Although this patient did not appear to have obvious visual abnormalities and the optic nerve was well preserved, previous reports on SPG11 patients have described decreased vision or visual evoked potential abnormalities [7, 18]. In addition, in a previous report on a Japanese patient with HSP-TCC who was not identified as having an *SPG11* mutation, degeneration of the LGB was described [1, 19]. Given that *SPG11* mutations are the most common type of mutation in patients with HSP-TCC in Japan, HSP with an *SPG11* mutation could involve the LGB, and LGB lesions should be considered a cardinal cause of visual abnormalities in patients with *SPG11* mutations. Taken together, these results suggest that SPG11 patients can present with LGB lesions, resulting in visual abnormalities.

We comprehensively evaluated the central nervous system of our patient and found p62-immunoreactive NCIs throughout, which corresponded to degenerated sites (Table 1). In contrast with the present patient, previously reported SPG11 patients showed limited distributions of p62-immunoreactive NCIs. Kuru et al. described eosinophilic granular inclusions in the substantia nigra, dentate nucleus, anterior horn, and spinal ganglia, but they did not confirm the nature of these inclusions using p62 immunostaining [14]. Denora et al. observed p62-immunoreactive eosinophilic granular inclusions in the anterior horn of the spinal cord, hypoglossal nucleus, and dorsal root ganglia [13]. Mori et al. observed p62-immunoreactive NCIs in the spinal cord and brainstem as well as in the subcortical nuclei and cingulate cortex; the patients reported in that study showed a somewhat broad distribution of p62-immunoreactive NCIs [20]. The present patient had a broader distribution of p62-immunoreactive NCIs and exhibited rapid clinical progression from onset until the need for nasal tube feeding, becoming bedridden and developing respiratory failure earlier than other autopsied patients with HSP-TCC and *SPG11* mutations (Table 2) [13, 14, 20]. These results raise the possibility that the widespread accumulation of p62-immunoreactive NCIs contributes to rapid clinical progression.

**Table 2:** Clinical characteristics of autopsied patients with hereditary spastic paraplegia with a thin corpus callosum and mutations in *SPG11*

Patient	Sex	Mutation of the *SPG11* gene	Age at death (years)	Age at onset of motor symptoms (years)	Time from motor symptom onset to clinical event (years)		
					Bedridden	Need for artificial ventilation	Death
Patient in Kuru, et al. (2005)	Male	#1 not examined in the patient	51	14	23	36	37
Patient 1 in Denora, et al. (2016)	Female	#2 in patient herself	32	12	Not described	Not needed	20
Patient 2 in Denora, et al. (2016)	Female	#3 not examined in the patient	46	10	< 35	Not needed	36
Patient 1 in Mori, et al. (2021)	Female	#4 in patient herself	57	25	15	Not needed	32
Present patient	Male	#5 in patient himself	44	13	12	13	31

#1 IVS18+1G>T developed in sister

#2 Two heterozygous truncating mutations; c.2358_2359delinsTT and c.4868delT

#3 A homozygous truncating mutation, c.6739_6742delGAGT, developed in brother

#4 Homozygous mutation, IVS18+1G>T

#5 Homozygous splice site variant, c.4162-2A>G

The patient also showed an accumulation of LBAS. The incidence of LBAS is known to increase with age [21], and LBAS is present in one-third of the aged population [22]. However, our patient showed LBAS, albeit mild, despite being in his mid-forties. Genetic analysis did not reveal any already known pathological genetic variations associated with Parkinson’s disease. Kuru et al. reported that the spatacsin protein, which is encoded by *SPG11*, is localized in Lewy bodies (LBs), Lewy neurites, and pale bodies. Pale bodies are considered to be precursors of LBs [23], therefore they hypothesized that spatacsin is involved in the early stages of LB formation. The accumulation of LBAS earlier in life in our patient may provide support for their hypothesis. *SPG11* mutations may accelerate the accumulation of LBAS. Future studies focusing on this hypothesis are expected.

In conclusion, we report a patient with spastic paraplegia with an *SPG11* mutation who showed p62-immunoreactive NCIs throughout the central nervous system that corresponded to degenerated sites. A variety of clinical phenotypes develop due to such widespread lesions. The LGB is also a site of degeneration in patients with SPG11. Consequently, detailed neurological examinations, including visual function assessments and wide-ranging neuropathological investigations, should be conducted in patients with SPG11 in the future. Furthermore, the comorbid LBAS pathology observed in the present patient suggests the need to further evaluate the involvement of *SPG11* in LB formation.

## Supplementary Information

**Additional file 1.** Methods of genetic analysis and neuropathological examination.

## Data Availability

The data that support the findings presented in this study are available from the corresponding author upon reasonable request.

## Abbreviations

ARHSP: Autosomal recessive hereditary spastic paraplegia
LB: Lewy body
LBAS: Lewy body-related α-synucleinopathy
LGB: Lateral geniculate body
NCI: Neuronal cytoplasmic inclusion
TCC: Thin corpus callosum
