# Supplementary material for: An autopsied case report of spastic paraplegia with thin corpus callosum carrying a novel mutation in the SPG11 gene: widespread degeneration with eosinophilic inclusions
Source: BMC Neurol. 2022 Jan 3;22:2. doi: 10.1186/s12883-021-02514-z (PMC8722294; doi:10.1186/s12883-021-02514-z)
Supplement: Supplementary file 1 — Additional file 1. Methods of genetic analysis and neuropathological examination. [file 12883_2021_2514_MOESM1_ESM.docx]

**Additional file 1**

**Genetic analysis**

For a definitive diagnosis, genetic counselling was provided, and written informed consent was obtained from the patient’s parents. The genetic analysis included a trio exome analysis performed to identify the genetic cause of the disease. Genomic DNA was extracted from peripheral blood samples of the patient and his parents. The TruSight One sequencing panels covering the coding regions of 4813 genes associated with a known clinical phenotype were run on a HiSeq instrument (Illumina, San Diego, USA). A mutation was detected around exon 25 of *SPG11*, and direct sequencing of *SPG11* (NM_025137.3) by the Sanger method at the same site was then performed.

**Neuropathological examination**

The brain and spinal cord were fixed in 20% buffered formalin and embedded in paraffin. Six-micrometer-thick sections were stained with haematoxylin-eosin and Klüver-Barrera and by Gallyas–Braak silver impregnation. Furthermore, immunoreaction product deposits on immunohistochemically stained sections were processed with a Ventana BenchMark GX autostainer (Ventana Medical Systems, Tucson, AZ, USA) and an I-View Universal DAB Detection Kit (Roche, Basel, Switzerland); the primary antibodies are listed in Additional Table 1. The specimen was investigated with a light microscope (Eclipse Ni, Nikon, Tokyo, Japan) and photographed using a digital camera (DS-Ri, Nikon, Tokyo, Japan). We semiquantitatively evaluated neuronal loss, gliosis, and p62-immunoreactive neuronal cytoplasmic inclusions (NCIs). Degeneration was assessed based on the degree of neuronal loss, indicated as absent (-), mild (+), moderate (++), severe (+++), or totally lost (++++), and gliosis was indicated as mild (+), moderate (++), or severe (+++). The p62-immunoreactive NCI frequency, evaluated with a 10× objective magnification, is indicated as follows: no NCI across the entire section (-), an average of 0–2 inclusions (+), an average of 3–9 inclusions (++), and an average of ≥ 10 inclusions (+++) per field.

**Additional Table 1: Antibody list**

| Primary antibodies | Host | Dilution | Source |
| --- | --- | --- | --- |
| p62 (3/P62 LCK LIGAND) | Mouse, monoclonal | 1:100 | BD Transduction Laboratories, Mountain View, CA, USA |
| Ubiquitin | Rabbit, polyclonal | 1:1000 | Dako, Glostrup, Denmark |
| Phosphorylated TDP-43 (pSer409/410) | Mouse, monoclonal | 1:10 000 | A gift from M. Hasegawa, Japan; now available for purchase from Cosmo Bio, Tokyo, Japan |
| Cystatin C | Rabbit, polyclonal | 1:10 000 | Dako, Glostrup, Denmark |
| Phosphorylated neurofilament (SMI31) | Mouse, monoclonal | 1:20 000 | Sternberger Monoclonals Inc., Baltimore, MA, USA |
| Phosphorylated tau (AT8) | Mouse, monoclonal | 1:1000 | Innogenetics, Ghent, Belgium |
| Human amyloid β 11-28 (12B2) | Mouse, monoclonal | 1:50 | IBL, Gunma, Japan |
| Phosphorylated α-synuclein (pSyn#64) | Mouse, monoclonal | 1:10 000 | A gift from T. Iwatsubo, Japan; now available for purchase from FUJIFILM Wako Pure Chemical Corporation, Osaka, Japan |
